# Impacts of the COVID-19 Pandemic on Intimate Partner Violence and Child Maltreatment Services in Rural Northwest North Carolina

**DOI:** 10.13023/jah.0603.08

**Published:** 2024-10-01

**Authors:** Elisabeth G. Galphin, Adam Hege, Amy Dellinger Page

**Affiliations:** University of North Carolina, Chapel Hill; Appalachian State University; Appalachian State University

**Keywords:** Appalachia, child maltreatment, COVID-19, intimate partner violence (IPV), public health emergencies, social service agencies

## Abstract

The current study examined the impact of the COVID-19 pandemic on IPV and child maltreatment services in rural northwestern North Carolina. Qualitative interviews were conducted with eight professionals representing six service organizations across four counties. The findings highlighted challenges these agencies faced throughout the pandemic, new risks for the clients served, and positive outcomes. In addition, it has been a useful learning experience as public health and social service agencies learn to serve their communities more effectively moving forward. This is especially relevant for rural communities, as it has put public health preparedness at the forefront.

## INTRODUCTION

The COVID-19 pandemic significantly impacted intimate partner violence (IPV) and child maltreatment services as well as the clients these agencies serve.^1^,^2^ Rates of IPV and child abuse, as well as the availability of and access to resources and services for survivors, often vary between rural and urban areas.^3^ Previous studies have shown that child maltreatment may also be higher in rural areas, largely due to socioeconomic challenges and increased rates of mental illness.^4^

The rural Northwestern part of North Carolina, in the Appalachian Mountains, is referred to as the High Country.^5^ The region includes eight counties, and like many rural areas, the COVID-19 pandemic greatly impacted this area. Across these counties, poverty rates are high (14–18%), uninsured rates are high (13–20%), unemployment rates are high (6.2–7.8%), and mental illness is higher than state and national averages; in addition, the population is largely white (~90%).^6^,^7^ Beyond just reported COVID-19 cases, the pandemic greatly impacted this area socially and economically, further increasing the risks of poverty, homelessness, and food insecurity. Due to the correlation of increased rates of IPV and child maltreatment within these circumstances^8^, the current study sought to understand the impacts on the services provided by local IPV and child maltreatment services in this region.

## METHODS

Qualitative interviews (N=8), involving six regional agencies (two violence shelters, the tri-county health department, social services, one school system, and the homeless shelter) were conducted by Zoom from January to March 2022 with professionals working in IPV and child maltreatment response and prevention in four of the High Country counties. This included one representative from each of the domestic violence shelters, two representatives from the tri-county, two from social services, one from the school system, and one from the homeless shelter. The 12 questions used to facilitate these interviews were developed using common themes from existing research and focused on the pandemic’s impact on the organization the services available, and the individuals or families served. The Appalachian State University Institutional Review Board deemed the study to be exempt from oversight.

## RESULTS

### Challenges Faced by Interviewed Agencies

One of the main challenges faced by all agencies was adapting and developing new and current protocols to adhere to state and federal COVID-19 guidelines. Telehealth, virtual meetings, and phone appointments increased significantly throughout the pandemic. Case management, counseling, classes, and even legal sessions moved to remote environments, which was new to both professionals and clients. Shifting to telehealth had both challenges and benefits.

New challenges and risk factors encountered by clients included increased isolation, financial barriers, chronic stress, the intensity of abuse, barriers to care, mental illness, and new experiences of abuse. Early in the pandemic, clients experienced less social support with isolation from loved ones and social programs temporarily stopping. Chronic stress from the uncertainty of COVID-19, increased financial strain, higher rates of unemployment, and being restricted to home more frequently were also burdens.

Many other barriers existed, including affordable and accessible housing due to the region being a resort area, transportation barriers, and a high prevalence of low-wage jobs. These barriers were exacerbated during the pandemic and increased stress for many residents. The lack of available resources (including reliable internet) and available shelter space in the area created further barriers for survivors of IPV.

### Positive Outcomes/Lessons Learned

Agencies were often able to expand their reach to better support and serve clients through new remote methods. Increased funding allowed the agencies to renovate shelters to make the area more welcoming for clients. Funding also enabled organizations to maintain housing programs through alternative methods, like hotels, and even adding more beds to their shelter.

The pandemic led to greater openness about mental health. Many people are now more open to discussing the impact of stress on mental well-being and learning about available services. Furthermore, there has been growth in the awareness of mental health needs for employees and how to reduce staff burnout. High Country agencies have begun implementing plans to better support staff, including remote working days.

The pandemic highlighted the resiliency of the agencies, staff, and local communities; residents of the High Country worked together to take care of one another and reduce the impacts of the pandemic on individuals and families. Professionals worked endlessly to eliminate barriers clients faced, provide needs, and continue to support them, all while juggling new roles COVID-19 brought on.

## IMPLICATIONS

The current study found many similarities in the experiences of the interviewed agencies and the clients served during the COVID-19 pandemic. Each agency altered how services and programs were provided and created new protocols for social distancing. Agencies also identified new risks and challenges clients faced during the pandemic. Some included being at home more often with their abuser, higher rates of chronic stress, economic hardships, increased isolation, and greater intensity of abuse. Finally, the organizations shared some positive outcomes. Professionals provided services in different ways, allowing some clients to feel more comfortable and supported while seeking assistance. Agencies also received more funding, enabling them to expand services and improve existing ones.

This study also highlighted policy gaps and need for future research and services. First, the pandemic highlighted needs for stronger emergency planning and management procedures, as well as greater funding to organizations. Greater focus on preparing for the next public health emergency would enable organizations and residents to feel more supported. Furthermore, prioritizing emergency preparedness would limit the impacts of the next crisis.

Greater attention must be given to establishing new legislation that protects all IPV and child maltreatment survivors, strengthening existing policies, and increasing funding to rural agencies. More comprehensive policies and programs are needed to equip individuals living in rural and low-income areas with the technology to have equal access to employment, healthcare, and school activities. Some examples of current policies include President Biden’s bipartisan Infrastructure Law which aims to provide internet access to all individuals in the U.S. and Governor Roy Cooper’s prioritization of resources to eliminate the digital divide in North Carolina.9,10 However, more policies are needed to ensure this technology is accessible to all citizens.
